# Polymorphisms of the *HTR2C* Gene as Predictors of Metabolic Disturbances During Clozapine Therapy: A Systematic Review and Meta-Analysis

**DOI:** 10.3390/jcm14113861

**Published:** 2025-05-30

**Authors:** Aiperi K. Khasanova, Dmitriy N. Sosin, Sergey N. Mosolov, Karin B. Mirzaev, Dmitriy A. Sychev

**Affiliations:** 1 Russian Medical Academy of Continuous Professional Education, 2/1, Bldg. 1, Barrikadnaya St., Moscow 125993, Russia; sosin.dmitriy@gmail.com (D.N.S.); profmosolov@mail.ru (S.N.M.); karin05doc@yandex.ru (K.B.M.); dmitry.alex.sychev@gmail.com (D.A.S.); 2 Moscow Research Institute of Psychiatry, Branch of the Serbsky National Medical Research Centre for Psychiatry and Narcology, 3, Poteshnaia St., Moscow 107076, Russia

**Keywords:** clozapine, genetic, *HTR2C* gene, metabolic adverse effects, obesity, polymorphism

## Abstract

**Background/Objectives:** Clozapine, the gold-standard treatment for treatment-resistant schizophrenia, is linked to metabolic disturbances such as weight gain and metabolic syndrome (MetS). **Methods:** This systematic review and meta-analysis evaluated the association between *HTR2C* polymorphisms and these adverse effects. Following PRISMA guidelines, 27 studies (n = 4044 patients, including 1804 clozapine-treated) were analyzed. **Results:** A meta-analysis revealed that the rs3813929 T allele was associated with a smaller increase in body weight, showing a mean BMI difference of 0.59 kg/m^2^ (95% CI: −1.02 to −0.17; *p* = 0.006), particularly in males. The rs1414334 C allele doubled MetS risk (OR: 2.15; 95% CI: 1.42–3.27; *p* = 0.0003). Haplotype analyses suggested combined genetic effects, though findings for other polymorphisms were inconsistent. Key limitations include study heterogeneity, small sample sizes, and the predominance of mixed antipsychotic regimens (clozapine with other psychotropics) in included studies, potentially confounding metabolic outcomes. Despite this, rs3813929 and rs1414334 emerge as promising pharmacogenetic markers for predicting metabolic risks. **Conclusions:** These results highlight the need for large-scale, prospective studies across diverse populations to validate associations and optimize personalized monitoring strategies. Implementing genetic screening could enhance early intervention, improving long-term outcomes for clozapine-treated patients.

## 1. Introduction

Despite advances in pharmacotherapy and the introduction of new antipsychotic agents, approximately one-third of patients with schizophrenia experience an insufficient treatment response, a condition known as treatment-resistant schizophrenia (TRS). In such cases, clozapine remains the gold standard, demonstrating unique efficacy against positive symptoms and being the only antipsychotic formally recommended for the management of TRS [1].

Nevertheless, despite its proven clinical benefits, clozapine use is frequently associated with a broad range of adverse effects, the most critical of which are metabolic disturbances. For instance, patients often experience weight gain, dyslipidemia, insulin resistance, and metabolic syndrome (MetS) during clozapine treatment [2,3,4]. The reported prevalence of overweight among clozapine-treated patients ranges from 35.7% to 80%; among patients treated with clozapine (mean age 45.3 ± 11.7 years), nearly 60% were diagnosed with MetS—a strikingly high prevalence for such a relatively young population, the majority of whom lived independently or with family [2]. According to the study by Lind, P.A. et al., weight gain was one of the most frequently reported adverse effects, affecting 71.0% of patients receiving clozapine [5]. This highlights the high prevalence of excess weight as a prominent metabolic concern among individuals receiving clozapine therapy. Dyslipidemia affects approximately 35% of clozapine users [6]. The prevalence of MetS among this population ranges from 40% to 60% [2]. A long-term naturalistic study conducted over more than two decades in the United States found that 42.7% of patients treated with clozapine developed diabetes mellitus [7].

MetS not only increases the risk of cardiovascular disease, type 2 diabetes mellitus, acute coronary syndrome, and cerebrovascular events, but also doubles the risk of mortality from cardiovascular causes and raises overall mortality by approximately 1.5–2 times [8,9].

Among patients with schizophrenia, metabolic disturbances represent a major contributor to the markedly reduced life expectancy observed in this population, estimated to be 15–20 years shorter than that of the general population [10].

The emergence of metabolic disturbances during clozapine treatment is a multifactorial process, involving genetic susceptibility as well as pharmacokinetic and pharmacodynamic factors [11]. Several studies have shown that patients experiencing a first episode of schizophrenia, even prior to antipsychotic exposure, already exhibit impaired glucose tolerance and insulin resistance, suggesting an intrinsically elevated risk of metabolic dysfunction independent of pharmacotherapy [12,13].

One of the principal genetic factors implicated in the metabolic effects of clozapine is the ***HTR2C*** gene, which encodes the serotonin 2C receptor (5-HT2C). This receptor plays a pivotal role in regulating appetite, energy homeostasis, and glucose and lipid metabolism [14]. Clozapine, a potent antagonist of 5-HT2C receptors, may exacerbate metabolic disturbances in part by disrupting this regulatory pathway [15].

Investigating ***HTR2C*** polymorphisms provides valuable insights into individual susceptibility to clozapine-induced metabolic side effects. Such knowledge is critical for advancing personalized treatment strategies, including dose optimization, targeted metabolic monitoring, and timely prevention and management of complications. This is particularly vital for patients with TRS, for whom clozapine remains the treatment of choice despite its associated metabolic risks.

To date, no systematic reviews or meta-analyses have comprehensively assessed the impact of different ***HTR2C*** polymorphisms on metabolic outcomes among clozapine-treated patients. To address this gap, we conducted a systematic review and meta-analysis to consolidate and critically appraise the existing evidence.

**Objective:** to assess the association between polymorphisms of the *HTR2C* gene and the risk of metabolic disturbances in patients receiving clozapine therapy.

## 2. Materials and Methods

This systematic review was conducted in accordance with PRISMA guidelines. The review protocol was registered with PROSPERO (registration number CRD42024563905) on 30 June 2024. We did not require ethical approval, as all included data were previously published and obtained in compliance with ethical standards following approval by the relevant regulatory authorities. We used RevMan 5.4.

### 2.1. Inclusion Criteria

We considered studies eligible if they investigated the role of *HTR2C* gene polymorphisms in the development of metabolic disturbances among patients treated with clozapine. Both observational and interventional studies were included, provided they contained genetic analyses of *HTR2C* and reported metabolic outcomes. Population: patients diagnosed with schizophrenia or other psychotic disorders, provided that individuals with schizophrenia were included in the sample. Treatment: Studies were eligible if patients received antipsychotic therapy involving clozapine. Preference was given to studies where a clozapine-treated subgroup could be identified or where clozapine comprised a significant proportion of the therapy. Exposure: *HTR2C* polymorphisms, identified according to international nomenclature (rsID). Outcomes: The presence of metabolic disturbances, including weight gain, central obesity, dyslipidemia, insulin resistance, and MetS. The diagnosis of MetS was based on the international criteria of National Cholesterol Education Program Adult Treatment Panel III [3] or the International Diabetes Federation [4]. Language: publications in English or Russian. Publication type: original, peer-reviewed research articles.

### 2.2. Exclusion Criteria

Studies were excluded if they lacked stratification by clozapine use, did not report *HTR2C* polymorphisms by rsID, or if associations with specific alleles could not be extracted (e.g., only haplotype data without allele-level resolution). Conference abstracts, reviews, dissertations, reports, and publications in non-peer-reviewed sources were also excluded, as were unpublished data (grey literature).

### 2.3. Search Strategy

Two reviewers (A. Khasanova and D. Sosin) independently conducted the search from 10 July to 15 September 2024.

Searches of the PubMed database were conducted using a combination of the following terms: (“genetic” OR “single nucleotide polymorphism”) AND (“metabolic syndrome” OR “METS” OR “obesity” OR “weight” OR “diabetes” OR “insulin” OR “glucose” OR “triglycerides” OR “cholesterol”) AND “clozapine” AND “HTR2C”.

Additionally, a search in Google Scholar was performed using the keywords: “HTR2C”, “clozapine”, “metabolic syndrome”, “diabetes”, “glucose”, and “obesity”.

Searches of Scopus, Web of Science, and Embase were not conducted due to limited institutional access.

Further searches in specialized databases, including PharmGKB (https://www.pharmgkb.org/ accessed on 16 August 2024), GeneCards (https://www.genecards.org/ accessed on 20 August 2024), NCBI Variation Viewer (https://www.ncbi.nlm.nih.gov/variation/view/ accessed on 31 August 2024), DGIdb (https://dgidb.org accessed on 6 September 2024), and the IUPHAR/BPS Guide to Pharmacology (https://www.guidetopharmacology.org accessed on 15 September 2024), were conducted specifically for the *HTR2C* gene.

After duplicate removal, initial screening was performed based on titles and abstracts. Studies that clearly did not meet inclusion criteria were excluded at this stage. Full-text versions of the remaining articles were subsequently assessed for eligibility. A snowballing approach was also employed, whereby reference lists of included articles were manually screened to identify additional relevant studies.

### 2.4. Quality Assessment

Risk of bias in the included studies was assessed using the Strengthening the Reporting of Genetic Association Studies (STREGA) checklist [16], as recommended for evaluating genetic association studies [17]. Each study was independently assessed by two reviewers; disagreements were resolved through discussion. No automation tools were used for risk of bias assessment. Quality assessment was independently conducted by two reviewers (K. Mirzaev and A. Khasanova).

### 2.5. Data Extraction and Study Grouping for Synthesis

Two reviewers independently extracted the data. For systematic presentation and comparability, studies were grouped according to the following parameters: (1) authors and year of publication, (2) study design, (3) sample size, (4) clinical population, (5) therapy details, (6) metabolic outcomes, (7) investigated *HTR2C* polymorphisms, and (8) study results. The extraction was independently conducted by two reviewers (A. Khasanova and D. Sosin).

### 2.6. Data Synthesis

Where possible, data from included studies were pooled in a meta-analysis using RevMan 5.4. The primary effect measure for dichotomous outcomes was the odds ratio (OR) with a 95% confidence interval (CI). For continuous outcomes (e.g., changes in body weight or body mass index [BMI]), the mean difference (MD) or standardized mean difference (SMD) were used, also with 95% CIs.

If direct effect estimates (e.g., OR or MD) or standard errors were not provided, they were manually calculated from reported 2 × 2 tables, mean values, standard deviations, confidence intervals, or *p*-values. Standard data transformations, such as recalculating mean differences from percentage changes and reconstructing confidence intervals in the absence of standard errors, were performed as needed. Correlation coefficient transformations (r-to-Z) were not applied, as correlation coefficients were not among the targeted effect measures.

Given the anticipated clinical and methodological heterogeneity among the included studies—such as differences in population characteristics, treatment regimens, genotyping methods, and metabolic outcome definitions—a random-effects model using the DerSimonian–Laird method was applied for all meta-analyses, regardless of the level of statistical heterogeneity. This approach aligns with best practices for meta-analyses of genetic associations, where true effect sizes are expected to vary across studies [17,18,19].

We assessed heterogeneity using the chi-squared (χ^2^) test for significance, the I^2^ statistic for the degree of inconsistency (low, moderate, high), and visual inspection of the forest plot. Sensitivity analyses were conducted to assess the robustness of meta-analytic results, including the addition of studies.

Publication bias was not formally assessed, as fewer than ten studies were included in each individual meta-analysis, rendering funnel plots and asymmetry tests (Egger’s, Begg’s) methodologically inappropriate.

Certainty of evidence was assessed using two complementary approaches. For outcomes subjected to meta-analysis, certainty was evaluated following the GRADE [20] framework, considering risk of bias, inconsistency, indirectness, imprecision, and publication bias. For outcomes not included in meta-analyses, formal GRADE assessment was not conducted. Instead, the strength of evidence was discussed narratively based on the methodological quality of the included studies, assessed using the STREGA checklist [16], and the general consistency or inconsistency of findings across studies. Certainty of evidence was assessed by two reviewers (S. Mosolov and D. Sychev). In cases of disagreement at any of the aforementioned stages, resolution was achieved through discussion; if consensus could not be reached, expert consultation was sought from S. Mosolov and D. Sychev.

## 3. Results

**Figure 1:**
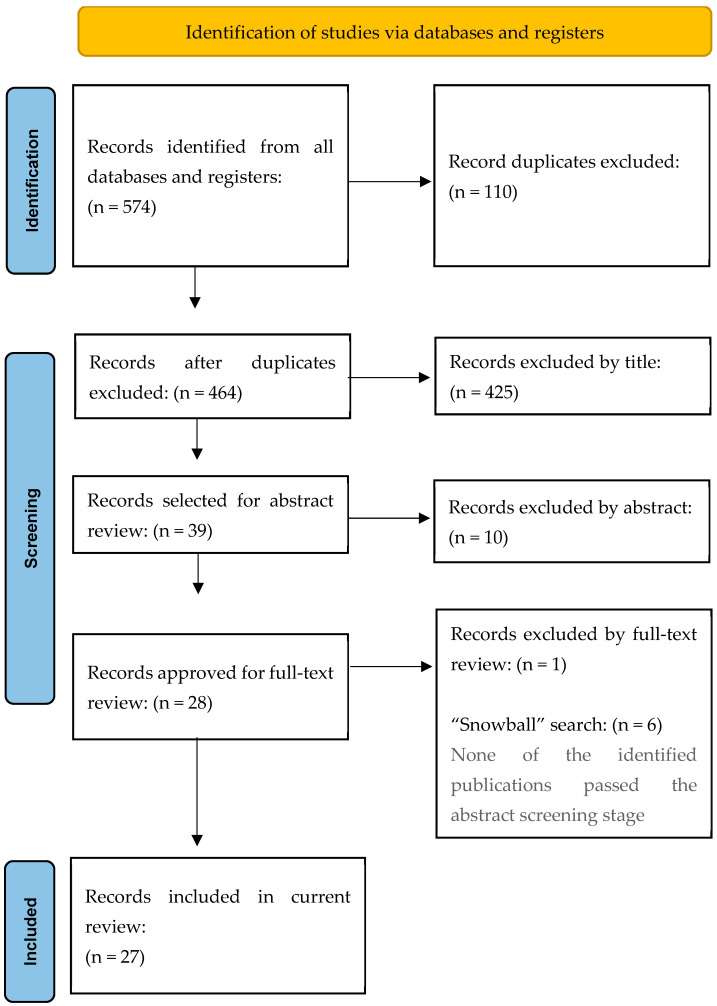
Flowchart of the literature search strategy.

The study selection process is summarized in Figure 1.

Our initial search yielded 574 original studies. After removing duplicates, we screened the remaining records based on titles and abstracts. Twenty-eight full-text articles were reviewed for eligibility. Ultimately, 27 studies met the inclusion criteria and were included in the systematic review.

The authors selected 27 studies for this systematic review, which included a total of 4044 patients with various psychiatric disorders. Diagnoses encompassed schizophrenia (SZ), schizoaffective disorder (SZA), delusional disorder (DD), schizophreniform disorder (SZF), schizophrenia spectrum disorders (SSDs), other psychotic disorders (PDs), mood disorders (MDs), treatment-resistant schizophrenia (TRS), and bipolar disorder (BD).

A total of 1804 patients received clozapine therapy, of whom 974 were treated with clozapine monotherapy. However, many studies did not specify whether clozapine was administered as monotherapy or as part of a combination regimen, rendering our estimates approximate. It was not possible to calculate the mean daily dose of clozapine, as dosing information was either not reported or incompletely documented in the majority of studies.

In addition to clozapine, participants were treated with other antipsychotics (APs), mood stabilizers (MDs), antidepressants (ADs), benzodiazepines, and sedative medications (SDs).

The studies were published between 1997 and 2019. Of the 27 studies: 17 employed a cross-sectional design, 2 utilized a case–control design, and 8 were prospective studies with observation periods ranging from 4 weeks to 6 months.

The studies were conducted in diverse populations, including Chinese, Korean, Thai, and Caucasian individuals (the latter group including Finnish, Dutch, and German participants), as well as Black, Hispanic, and mixed-ethnicity cohorts. Some studies also classified participants broadly as “Asian”, “White”, “Black”, or “Mixed”. Populations specifically described as “Turkish”, “Hindu”, or “Mediterranean” were not reported within the included samples.

We categorized the 27 studies into three groups based on their primary metabolic outcomes:

1. Association of genetic polymorphisms with weight gain (Table 1);
2. Association with metabolic syndrome (MetS) (Table 2);
3. Association with other metabolic outcomes (Table 3).

Across the 27 included studies, a total of 14 genetic variants were investigated: rs3813929 (–759C>T), rs3813928 (–759C>T) or (997G>A), rs1414334 (C>G), rs498207, rs6318 (Cys23Ser) or G68C, (GT)12–18/(CT)4–5, HTR2C:c.1–142948(GT)n, rs518147 (697G>C), rs12836771 (A>G), rs521018, rs498177, rs2192371, rs5988072, and rs12833104. Overall, the reporting quality of the included studies was good according to the STREGA (Strengthening the Reporting of Genetic Association Studies) [47] criteria, with an average compliance rate of 76.4% (±13.6%). (Rietschel et al. (1997) [21] ≈ 80.6%, Hong et al. (2001) [22] ≈ 53.0%, Tsai et al. (2002) [25] ≈ 50.0%, Basile et al. (2002) [23] ≈ 50.0%, Reynolds et al. (2002) [24] ≈ 59.0%, Reynolds et al. (2003) [26] ≈ 59.0%, Theisen et al. (2004) [27] ≈ 83.3%, Miller et al. (2005) [28] ≈ 62.0%, Mulder et al. (2007) [29] ≈ 89.0%, De Luca et al. (2007) [17] ≈ 58.0%, Mulder et al. (2007) [39] ≈ 77.0%, Yevtushenko et al. (2008) [40] ≈ 69.4%, Gunes et al. (2009) [31] ≈ 55.0%, Mulder et al. (2009) [41] ≈ 82.0%, Popp et al. (2009) [30] ≈ 60.5%, Gregoor et al. (2010) [33] ≈ 72.0%, Opgen-Rhein et al. (2010) [32] ≈ 82.0%, Gregoor et al. (2011) [34] ≈ 68.0%, Kang et al. (2011) [44] ≈ 61.0%, Bai et al. (2011) [43] ≈ 82.0%, Kang et al. (2014) [35] ≈ 80.0%, Klemettilä et al. (2015) [36] ≈ 63.0%, Vasudev et al. (2017) [37] ≈ 83.3%, Rico-Gomis et al. (2016) [45] ≈ 91.7%, Puangpetch et al. (2018) [46] ≈ 59.0%, Puangpetch et al. (2019) [38] ≈ 64.1%; Risselada et al. (2010) [42] ≈ 96%.)

### 3.1. Weight Gain

Analysis of the scientific literature investigating the association between the **rs3813929 (–759C>T)** polymorphism and weight gain during clozapine treatment revealed that, among 17 studies, only three [24,26,28] identified an association between the 759T allele and a smaller increase in body weight. In two of these studies, the protective effect was particularly pronounced in male patients, but among them, one study in women found no association. In one study [23], an inverse relationship was observed, with the T allele being more frequent among patients who experienced weight gain; however, the primary analysis was conducted at the haplotype level. Gregoor et al. (2011) [34] and Kang et al. (2014) [35] reported a trend toward a protective effect of the 759T allele, although it did not reach statistical significance.

Despite the lack of significant associations for rs3813929 in most studies, several investigations demonstrated significant findings at the haplotype level. De Luca et al. (2007) [17] described a protective effect of the long-C-Ser haplotype. Gregoor et al. (2010) [33] reported that the combination of the LEP –2548G allele and the absence of the T allele was associated with obesity. Opgen-Rhein et al. (2010) [32] identified the AGC haplotype (A in rs498207, G in rs3813928, C in rs3813929) as being more frequent in patients with weight gain, whereas the GAT haplotype was more common among those without weight gain. Gunes et al. (2009) [31] found that homozygosity for the *HTR2C* haplotype (759C, 697G, Cys23) was associated with obesity. Mulder et al. (2007) [29] demonstrated that the combination of the HTR2C:c.1–142948(GT)n 13 allele, and the rs3813929 C, rs518147 C, and rs1414334 C alleles was associated with an increased risk of obesity.

Thus, across most studies examining combined genotypes and haplotypes, there is evidence suggesting a protective role for the 759T allele of rs3813929 against weight gain, particularly in males.

The **rs6318 (Cys23Ser)** polymorphism was evaluated in six studies, none of which found a significant association with weight gain. Nevertheless, Gunes et al. (2009) [31] reported that homozygosity for the *HTR2C* haplotype (759C, 697G, Cys23) was associated with obesity during clozapine treatment, whereas De Luca et al. (2007) [17] described a protective effect of the long-C-Ser haplotype (extending from 759C to Ser23) against weight gain.

The **rs1414334 (551–3008 C>G)** polymorphism was investigated in four studies [25,33,37,41]. In one study [41], the C allele was associated with obesity, particularly within a haplotype including rs3813929 C, rs518147 C, and (GT)n 13. Vasudev et al. (2017) [37] found that the G allele was associated with lower BMI in males. The other two studies did not detect significant associations; in one of them [45], the observed trend was in the opposite direction but did not reach statistical significance [33].

Regarding the **rs518147 (697G>C)** polymorphism, it was studied in two reports [29,46]. Mulder et al. (2007) [29] found an association with weight gain only within a combined genotype: carriers of the rs1414334 C, rs518147 C, (GT)n 13, and rs3813929 C alleles exhibited an increased risk of obesity, whereas Puangpetch et al. (2019) [38] found no significant association.

The **HTR2C:c.1–142948(GT)n** polymorphism was investigated in two studies. Mulder et al. (2007) [29] found that carriage of the 13-repeat allele was associated with obesity, whereas Gregoor et al. (2010) [33] did not replicate this finding.

Only one study [32] examined the **rs498207 (1165A>G)** polymorphism and found that the A allele and the AGC haplotype (A in rs498207, G in rs3813928, C in rs3813929) were more frequent among patients who experienced weight gain, while the GAT haplotype was more common among those who did not.

The **rs3813928 (997G>A)** polymorphism was investigated in a single study [29], which did not find a significant association.

### 3.2. Metabolic Syndrome

The most extensively studied polymorphism associated with the development of metabolic syndrome (MetS) during clozapine treatment is **rs1414334 (C>G)**. Among five studies investigating this variant, three [29,41,42] confirmed that carriage of the C allele increases the risk of developing MetS. Two other studies [33,35] found no significant association. Mulder and colleagues, in two separate studies [39,41], demonstrated that combined genotypes involving the HTR2C:c.1–142948(GT)n 13R allele, rs3813929 C, rs518147 C, and rs1414334 C were also associated with MetS.

Three studies explored the role of the **rs518147 (697G>C)** polymorphism. Significant associations between the C allele and MetS were reported in two studies [29,46], whereas Kang et al. (2011) [44] found no such association.

The **rs3813929 (759C>T)** polymorphism was investigated in three studies [29,41,42], none of which found a significant association with MetS outside of haplotype analyses.

Similarly, three groups evaluated the **rs3813928 (997G>A)** polymorphism; none detected a significant association with MetS, even when haplotypic analyses were performed [29,41,42].

The **HTR2C:c.1–142948(GT)n** polymorphism was studied in two reports. Mulder et al. (2007) [29] found that the 13-repeat allele was associated with MetS, particularly within a haplotype including rs3813929 C, rs518147 C, and rs1414334 C. However, Gregoor et al. (2010) [33] did not confirm this finding.

The **rs12836771 (A>G)** polymorphism, a tag-SNP for rs3813929, demonstrated a positive association with MetS in one study [46]. No additional studies have been identified.

The **rs498177 CC** genotype was associated with an increased risk of MetS in women, and the rs521018–rs498177 haplotype was more frequent among individuals with MetS [43]. No additional studies have been identified.

Polymorphisms **rs521018, rs2192371, rs5988072, rs12833104, rs6318, and rs498177** were investigated in relation to MetS in only one study [43], with none showing significant associations.

### 3.3. Other Metabolic Disturbances

Twelve studies reported associations between *HTR2C* gene variants (rs3813929 [–759C/T], rs3813928 [–997G/A], rs518147 [–697G/C], rs1414334 [c.551-3008 C>G], HTR2C:c.1–142948(GT)n 13R, rs6318 [Cys23Ser], rs521018, rs498177, rs2192371, rs5988072, rs12833104, and rs12836771 [A/G]), and other metabolic disturbances not mentioned above [31,36,37,38,39,40,41,42,43,44,45,46].

The **rs1414334** polymorphism was investigated in six studies. Two studies found that the rs1414334 C allele was associated with higher waist circumference (WC) [39,41], while no association was found in Risselada et al. (2010) [42], Klemettilä et al. (2015) [36], and Vasudev et al. (2016) [37].

Two studies [41,42] reported that the rs1414334 C allele was positively associated with hypertriglyceridemia, whereas Klemettilä et al. (2015) [36] reported that the GG genotype of rs1414334 was associated with higher triglyceride (TG) levels. No association between rs1414334 and TG levels was found in Mulder et al. (2007) [39] or Vasudev et al. (2017) [37].

Conflicting findings were also noted for HDL cholesterol. Risselada et al. (2012) [42] reported that the rs1414334 C allele showed a trend toward lower HDL cholesterol, whereas Klemettilä et al. (2015) [36] found that the GG genotype of rs1414334 was associated with lower HDL cholesterol. No associations between rs1414334 and HDL cholesterol levels were reported in Rico-Gomis et al. (2016) [45], Vasudev et al. (2017) [37], Mulder et al. (2009) [41], or Mulder et al. (2007) [39].

Mulder et al. (2007) [39] found a positive association between the **rs518147** (–697C) allele and higher WC. However, no associations were found in later studies [31,41,44,46].

In addition, Mulder et al. (2007) [39] reported that the **HTR2C:c.1–142948(GT)n 13R** allele was associated with higher WC, although this finding was not replicated by the same research group in their 2009 study [41].

Yevtushenko et al. (2008) [40] found that the combination of the ***HTR2C* (–759C/T) CC** genotype and the ***LEP* (–2548A/G) G** allele was positively associated with higher WC.

No significant associations were identified for the remaining polymorphisms in relation to the other metabolic disturbances studied.

## 4. Meta-Analysis

### 4.1. rs3813929 (–759C/T) and Weight Gain

**Figure 2:**
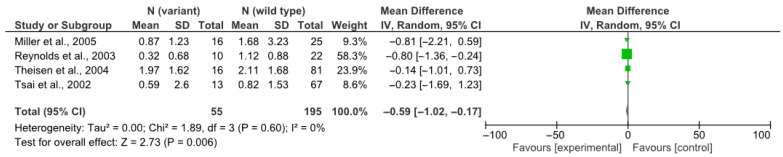
Primary meta-analysis: rs3813929 (–759C/T) and weight gain [25,26,27,28].

The primary meta-analysis included only those studies that met all of the following criteria: patients were treated with clozapine monotherapy (without concomitant use of other antipsychotics); data on changes in body mass index (ΔBMI) were presented as mean values (Mean) and standard deviations (SDs) for the genotype groups being compared; the sample size, including the number of carriers of each allele, was clearly reported; and where necessary, ΔBMI could be accurately approximated based on the percentage change in body weight, provided that baseline BMI and SD values were available (Figure 2).

**Figure 3:**
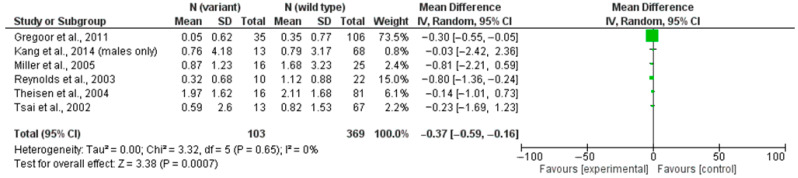
Sensitivity analysis: rs3813929 (–759C/T) and weight gain [25,26,27,28,34,35].

The sensitivity analysis included studies that did not meet one or more of the above criteria but provided partially comparable data. This group included: studies in which patients received clozapine in combination with other psychotropic medications, provided that patients on clozapine comprised more than 35% of the sample; studies lacking complete quantitative data on the sample (e.g., data reported only for male patients); and publications where ΔBMI was approximated from changes in body weight (in kilograms) under the assumption of a standard height (e.g., 1.70 m) for calculation purposes (Figure 3).

A meta-analysis of four studies (n = 250) [25,26,27,28] demonstrated that carriage of the T allele of the rs3813929 polymorphism was associated with a smaller increase in body mass index (ΔBMI) among patients receiving clozapine, compared to carriers of the wild-type CC genotype. The pooled mean difference was 0.59 kg/m^2^ (95% CI: −1.02 to −0.17; *p* = 0.006). Heterogeneity analysis revealed no statistically significant differences between studies, indicating a high degree of consistency across the results. Despite the absence of heterogeneity, a random-effects model was employed to account for potential underlying variability.

The extended sensitivity analysis included studies by Gregoor et al. (2011) [34], Kang et al. (2014) (male subgroup) [35], and Basile et al. (2002) [23], in addition to the studies from the primary analysis pool. The pooled mean difference in the sensitivity analysis was −0.37 kg/m^2^ (95% CI: −0.59 to −0.16; *p* = 0.0007), confirming a statistically significant protective effect of the T allele against BMI increase. Even when including studies with less stringent quality criteria, no heterogeneity was observed, supporting the stability of the effect. These results reinforce the robustness of the primary finding that carriers of the rs3813929 T allele experience a smaller ΔBMI increase during clozapine treatment.

### 4.2. rs1414334 (C>G) and Metabolic Syndrome (MetS)

**Figure 4:**
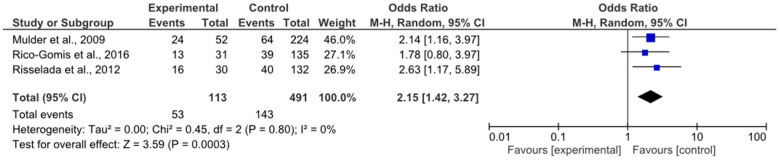
rs1414334 (C>G) and metabolic syndrome (MetS) [41,42,45].

The meta-analysis included studies in which patients were treated with clozapine as well as other atypical antipsychotics. Both studies involving clozapine monotherapy and studies involving combination antipsychotic therapies were eligible. The primary outcome was the presence of MetS, diagnosed according to international criteria (NCEP ATP III or IDF). Studies were included if they reported odds ratios (ORs) with confidence intervals (CIs), or if genotype-specific frequencies of MetS were provided, sufficient to manually calculate ORs (e.g., via 2 × 2 contingency tables). In this meta-analysis, inclusion criteria were broadened because only one study investigated patients receiving clozapine monotherapy (Figure 4).

A meta-analysis of three studies [41,42,45] evaluating the association between the *HTR2C* rs1414334 (C>G) polymorphism and the risk of developing MetS revealed a statistically significant association between carriage of the C allele and an increased likelihood of MetS. The pooled odds ratio was 2.15 (95% CI: 1.42–3.27; *p* = 0.0003), indicating an almost twofold increased risk compared to G allele carriers. The absence of heterogeneity (I^2^ = 0%) highlights the high degree of consistency among studies and strengthens confidence in the reproducibility of the observed effect.

## 5. Discussion

In this systematic review and meta-analysis, data from 27 studies investigating the association between *HTR2C* gene polymorphisms and the development of metabolic disturbances in patients receiving clozapine therapy were analyzed.

In total, data from 4044 patients with various psychiatric disorders—primarily schizophrenia and schizoaffective disorder—were included.

The analysis of the rs3813929 (–759C/T) polymorphism and weight change during clozapine treatment demonstrated that carriage of the T allele was associated with a smaller increase in body mass index (ΔBMI). Although the protective effect of the T allele did not reach statistical significance in most individual studies, the pooled meta-analytic results confirmed a significant protective effect, with no evidence of heterogeneity across studies. This association appeared particularly pronounced in male subgroups.

The investigation of the rs1414334 (C>G) polymorphism revealed that carriage of the C allele was significantly associated with an increased risk of developing MetS. A meta-analysis of three studies showed nearly a twofold increase in MetS risk among C allele carriers compared to G allele carriers. The absence of heterogeneity further strengthens confidence in the validity of this association.

Single studies, without independent replication, suggested associations between the rs12836771 and rs498177 polymorphisms and the development of MetS.

Isolated studies also indicated possible associations of rs1414334 (551–3008 C>G), rs518147, and HTR2C:c.1–142948(GT)n polymorphisms with weight gain; however, findings remained inconsistent. Similar inconsistencies were observed regarding the associations of rs518147, rs3813928, and HTR2C:c.1–142948(GT)n with MetS.

For other metabolic disturbances, results across studies were also contradictory.

Some studies suggested that specific allele combinations were associated with metabolic outcomes: The combination of HTR2C:c.1–142948(GT)n 13R, rs518147 C, rs3813929 C, and rs1414334 C was associated with an increased risk of obesity. The same combination, excluding rs3813929 C, was linked to an increased risk of MetS. The long–C–Ser haplotype was associated with a reduced risk of weight gain, while the AGC haplotype (rs498207 A, rs3813928 G, rs3813929 C) was linked to increased weight gain. Homozygosity for the 759C, 697G, and Cys23 haplotype was associated with obesity. The combination of absence of the –759T allele and presence of the LEP –2548G allele was associated with an increased risk of obesity and higher waist circumference WC. However, these findings are based on isolated studies without independent replication.

Our findings highlight the potential clinical relevance of analyzing *HTR2C* genetic variations when planning clozapine therapy. In cases of identified genetic susceptibility, enhanced monitoring of metabolic parameters and early preventive interventions—such as initiating combination therapy with clozapine and agents like metformin or aripiprazole—may be considered to minimize metabolic side effects. Nevertheless, at the current stage, routine genetic testing is limited by the lack of reproducibility in available data and the absence of standardized clinical guidelines. Thus, caution is warranted when interpreting genetic results for decision making in therapy initiation or modification. Future research should focus on large-scale, prospective, ethnically stratified cohort studies with standardized assessments of metabolic outcomes and clozapine dosing. Particular emphasis should be placed on replicating the observed associations and developing comprehensive risk models integrating genetic, clinical, and behavioral factors. The creation of validated polygenic risk scores could become a promising tool for integrating genetic data into real-world clinical practice, but strict validation in independent cohorts is essential before clinical implementation.

### 5.1. Certainty of Evidence

For outcomes subjected to meta-analysis, we assessed the certainty of evidence using the GRADE framework [20]. The certainty of evidence for the association between rs3813929 (–759C/T) and weight gain was rated as **moderate**, based on the consistent results across studies, low risk of bias, and precise confidence intervals. The certainty of evidence for the association between rs1414334 (C>G) and metabolic syndrome was rated as low, primarily due to indirectness arising from the inclusion of studies where patients were treated with clozapine in combination with other antipsychotics. For outcomes and polymorphisms not included in quantitative syntheses, formal GRADE assessment was not conducted. Instead, the strength of evidence was discussed narratively, based on the risk of bias (assessed by the STREGA checklist) and the overall consistency or inconsistency of findings across the included studies.

### 5.2. Limitations

Despite the findings presented, this review has several important limitations: There was considerable heterogeneity among the included studies, both in terms of population characteristics (ethnicity and clinical profiles) and in the duration of follow-up. A limited number of studies involved patients on clozapine monotherapy; most received combination therapy with other antipsychotics, reducing the specificity of findings for clozapine. Variability in data reporting was observed, with several studies lacking direct effect measures (e.g., ORs or ΔBMI) or sufficient genotype-specific information, which prevented their inclusion in meta-analyses and necessitated manual recalculations in some cases. Particularly for rs1414334 and MetS, the included studies involved patients treated with multiple antipsychotics, potentially introducing indirectness. Although several potentially relevant studies were excluded due to missing numerical data, the consistency of findings among included studies supports the robustness of the results. Limitations also include the inability to fully account for potential effect modifiers such as sex, ethnicity, baseline weight, diet, and physical activity, and the small number of studies per meta-analysis (n < 10), which precluded formal evaluation of publication bias. Furthermore, many included studies had relatively small sample sizes, and information regarding clozapine dosing, duration of therapy, and concurrent risk factors was often incomplete, limiting the ability to perform more detailed stratified analyses.

## 6. Conclusions

Our review highlights the promise of *HTR2C* genetic variation as a pharmacogenetic marker for clozapine-induced metabolic disturbances.

The rs3813929 and rs1414334 polymorphisms emerged as the most consistently replicated and potentially clinically relevant variants. Further investigation of these polymorphisms—both individually and within haplotypes—could support the development of personalized strategies for monitoring, preventing, and managing the metabolic side effects of antipsychotic therapy.

Large, multicenter, prospective studies, taking into account clinical and genetic interactions as well as individual patient characteristics (e.g., sex, ethnicity, lifestyle), are necessary to confirm and refine these findings.

## Figures and Tables

**Table 1:** Association of genetic polymorphisms with weight gain.

Authors	Study Design	Sample	Population	Therapy	Polymor- Phisms	Results
Rietschel et al., 1997 [21]	Cross-sectional (AP ≥ 28 days)	152 SZ, SZA, PD	Europeans	MONO CLOZ	rs6318 (Cys23Ser)	No association
Hong et al., 2001 [22]	Prospective (4 months)	93 TR SZ	Europeans	MONO CLOZ	rs6318 (Cys23Ser)	No association
Basile et al., 2002 [23]	Prospective (6 weeks)	80 SZ	Europeans and African Americans	MONO CLOZ	rs3813929 (–759C/T)	Hemizygous T allele men gainedmore weight thanhemizygous C allele men
Reynolds et al., 2002 [24]	Prospective (10 weeks)	123 FE SZ	Chinese	CLOZ-4, AP	rs3813929 (–759C/T)	T-allele carriers gained less weight
Tsai et al., 2002 [25]	Prospective (4 months)	80 SZ, SZA	Chinese	MONO CLOZ	rs3813929 (–759C/T)	No association
Reynolds et al., 2003 [26]	Prospective (6 weeks)	32 SZ	Chinese	MONO CLOZ	rs3813929 (–759C/T)	Men with T allele gained less weight
Theisen et al., 2004 [27]	Prospective (12 weeks)	97 SZ	Europeans	MONO CLOZ	rs3813929 (–759C/T)	No association
Miller et al., 2005 [28]	Prospective (6 months)	41 TR SZ	Caucasian, African American, Hispanic	MONO CLOZ	rs3813929 (–759C/T)	T-allele carriers gained less weight
Mulder et al., 2007 [29]	Cross-sectional (AP ≥ 3 months)	127 SZ, SZA, PD	Europeans	CLOZ-44, AP PHC	rs3813929 (–759C/T), rs3813928 (–997G/A), rs518147 (–697G/C), rs1414334 (C>G), HTR2C c.1–142948(GT)n 13R	Carriers of the combined genotype rs1414334 C, rs518147 C, (GT)n 13, and rs3813929 C, and carriers of s1414334 C are related to an increased risk of obesity
De Luca et al., 2007 [17]	Prospective (6–14 weeks)	139 SZ	various ethnicities, including African Americans	CLOZ-92, AP	rs3813929 (–759C/T), rs6318 (Ser23Cys), (GT)12–18/(CT)4–5	The Long-C-Ser haplotype confers a protective effect against weight gain
Popp et al., 2009 [30]	Prospective (4 weeks)	102 SZ, SZA, PD	Europeans	CLOZ-16, AP, AD, SD, NT, and PHC	rs6318 (Cys23Ser)	No association
Gunes et al., 2009 [31]	Cross-sectional (AP ≥ 6 months months)	46 SZ, SZA	white origin	CLOZ-18, OL	rs3813929 (–759C/T), rs518147 (–697G/C), rs6318 (Cys23Ser)	Haplotype (759C, 697G, 23Cys) is associated with an increased risk of obesity
Opgen-Rhein et al., 2010 [32]	Retrospective case–control (AP ≥ 6 weeks)	128 SZ	Europeans, Turks	CLOZ-24, AP, AD, NT, and PHC	rs498207 (–1165A/G), rs3813928 (–997G/A), rs3813929 (–759C/T), rs6318 (Cys23Ser)	Weight gain was higher in hemizygous males carrying the rs498207 A allele and in females with the rs498207 AA genotype, as well as in carriers of rs3813928 and rs3813929. The AGC haplotype (rs498207 A, rs3813928 G, rs3813929 C) is associated with increased weight gain
Gregoor et al., 2010 [33]	Cross-sectional (AP ≥ 3 months)	200 SZ, SZA, PD	Europeans	CLOZ-67, AP, PHC	rs3813929 (–759C/T), rs1414334 (c.551-3008 C>G)	The combination of the absence of the –759T allele and the presence of the LEP –2548G allele is associated with an increased risk of obesity
Gregoor et al., 2011 [34]	Prospective (per year)	141 PSD, AFD, PD	Europeans	CLOZ-68, AP, PHC	rs3813929 (–759C/T)	The HTR2C –759T allele exhibited a trend toward protection against weight gain
Kang et al., 2014 [35]	Retrospective (AP ≥ 1 year)	113 SZ	Koreans, Chinese	CLOZ MONO-68, AP, NT, PHC	rs3813928 (–759C/T)	The HTR2C –759T allele exhibited a trend toward protection against weight gain in men
Klemettilä et al., 2015 [36]	Cross-sectional (AP ≥ 1 year)	190 SZ	Europeans	CLOZ MONO-121, CLOZ + AP-69	rs1414334 (c.551-3008 C>G)	No association
Vasudev et al., 2017 [37]	Cross-sectional (AP ≥ 6 months)	60 SZ, SZA, DD	overwhelming majority Europeans, First Nations	CLOZ-60, CLOZ MONO-18, CLOZ + AP/AD/NT	rs3813929 (–759C/T), rs3813928 (–697G/A), rs1414334 (c.551-3008 C>G)	The *HTR2C* rs1414334 G allele is associated with weight loss in men
Puangpetch et al., 2019 [38]	Cross-sectional (AP ≥ 1 year)	180 SZ	Thais	CLOZ-50, AP	rs518147 (–697 G/C), rs12836771 (A/G)—tag-SNP for rs3813929 (–759C/T)	No association

**Table 2:** Association of genetic polymorphisms with metabolic syndrome (MetS).

Authors	Study Design	Sample	Population	Therapy	Subject of Study	Polymorphisms	Results
Mulder et al., 2007 [39]	Cross-sectional (AP ≥ 3 months)	112 SZ, SZA, PD	White	CLOZ-41, AP, and PHC	MetS (NCEP: ATP III)	rs3813929 (–759C/T), rs3813928 (–997G/A), rs518147 (–697G/C), rs1414334 (C>G), HTR2C c.1–142948(GT)n 13R	rs1414334 C, rs518147 C, (GT)n 13R are associated with an increased risk of MetS; strong positive association of the combined haplotype (especially in men) (13 repeat + rs518147 C + rs1414334 C) with MetS
Yevtushenko et al., 2008 [40]	Cross-sectional (AP long)	134 SZ, SZA	Europeans	CLOZ MONO-21, AP	MetS (IDF)	rs3813929 (–759C/T)	The combination of the *HTR2C* C allele and the LEP G allele is positively associated with MetS
Mulder et al., 2009 [41]	Cross-sectional replication (AP ≥ 3 months), pooled analysis	164 in replication sample, 276 in pooled analysis	Asian, African, Mediterranean, Hindustan origins	CLOZ-37 (replication sample), AP, PHC	MetS (NCEP: ATP III)	rs3813929 (–759C/T), rs518147 (–697G/C), rs1414334 (c.551-3008 C>G), HTR2C c.1–142948(GT)n 13R	The rs1414334 C allele showed a trend toward positive association with MetS in the replication cohort. In the combined analysis, both rs1414334 C and the HTR2C c.1–142948(GT)n 13R variant are associated with an increased risk of MetS
Risselada et al., 2012 [42]	Cross-sectional replication (AP long)	186 SZ, SZA, PD	Overwhelmingly Europeans	CLOZ-31, AP, PHC	MetS (NCEP: ATP III)	rs3813929 (–759C/T), rs1414334 (c.551-3008 C>G)	The rs1414334 C allele is associated with an increased risk of MetS
Bai et al., 2011 [43]	Case–control study (AP ≥ 3 months)	456 SZ	Chinese	CLOZ MONO-171, AP	MetS (IDF Asia)	rs521018, rs498177, rs2192371, rs5988072, rs12833104, rs6318 (Cys23Ser)	The rs498177 CC genotype is associated with an increased risk of MetS in women, and the rs521018–rs498177 haplotype is more frequent among individuals with MetS
Kang et al., 2011 [44]	Cross-sectional (AP ≥ 1 year)	146 SZ	Korean	CLOZ-146, CLOZ + AP, CLOZ + NT	MetS (NCEP ATP IIIA Asia)	rs3813928 (–759C/T), rs518147 (–697G/C)	No association
Vasudev et al., 2017 [37]	Cross-sectional (AP ≥ 6 months)	60 SZ, SZA, DD	Overwhelmingly Europeans, First Nations	CLOZ-60, CLOZ MONO-18, CLOZ + AP/AD/NT	MetS (NCEP: ATP III)	rs3813929 (–759C/T), rs3813928 (–697G/A), rs1414334 (c.551-3008 C>G)	No association
Rico-Gomis et al., 2016 [45]	Cross-sectional (AP ≥ 3 months)	166 SZ, SZA, SZF, PD, BD	Europeans	CLOZ, AP	MetS (IDF)	rs1414334 (c.551-3008 C>G)	No association
Puangpetch et al., 2018 [46]	Cross-sectional (AP long)	113 SZ	Thais	CLOZ-47, AP, PHC	MetS (IDF)	rs518147 (–697G/C), rs12836771 (A/G)—tag-SNP for rs3813929 (–759C/T)	The rs518147 CC and rs12836771 GG genotypes are associated with an increased risk of MetS

**Table 3:** Association of genetic polymorphisms with other metabolic outcomes.

Authors	Study Design	Sample	Population	Therapy	Subject of Study	Polymorphisms	Results
Mulder et al., 2007 [39]	Cross-sectional (AP ≥ 3 months)	112 SZ, SZA, PD	White	CLOZ—41, AP, and PHC	WC, TG, HDL, BP	rs3813929 (–759C/T), rs3813928 (–997G/A), rs518147 (–697G/C), rs1414334 (C>G), HTR2C:c.1–142948(GT)n 13R	The HTR2C:c.1—142948(GT)n 13R, rs518147 (–697) C, rs1414334 C alleles are associated with higher WC
Yevtushenko et al., 2008 [40]	Cross-sectional (AP ≥ 3 months)	134 SZ, SZA	Europeans	CLOZ MONO—21, AP	WC, TG, HDL, BP, GC	rs3813929 (–759C/T)	The combination of the HTR2C (–759C/T) CC genotype and the LEP (72548A/G) G allele is positively associated with higher WC
Mulder et al., 2009 [41]	Cross-sectional (AP long)	164 in replication sample, 276 in pooled analysis	Asian, African, Mediterranean, and Hindustan origins	CLOZ—37 (replication sample), AP, PHC	WC, TG, HDL, BP	rs3813929 (–759C/T), rs518147 (–697G/C), rs1414334 (c.551-3008 C>G), HTR2C:c.1–142948(GT)n 13R	rs1414334 C allele has a positive association with hypertriglyceridemia and higher WC
Gunes et al., 2009 [31]	Cross-sectional replication (AP ≥ 3 months), pooled analysis	46 SZ, SZA	white origin	CLOZ—18, OL	WG, insulin, C-peptide, TG, cholesterol, HOMA-IR	rs3813929 (–759C/T), rs518147 (–697G/C), rs6318 (Cys23Ser)	No association
Risselada et al., 2010 [42]	Cross-sectional (AP ≥ 6 months)	186 SZ, SZA, PD	Overwhelmingly Europeans	CLOZ-31, AP, PHC	WC, TG, HDL, BP, GC	rs3813929 (–759C/T), rs1414334 (c.551-3008 C>G)	The rs1414334 C allele is positively associated with increased TG. There was a trend toward lower HDL cholesterol associated with the rs1414334 allele
Bai et al., 2011 [43]	Cross-sectional replication (AP long)	456 SZ	Chinese	CLOZ MONO-171, AP	TG, HDL, BP, GC	rs521018, rs498177, rs2192371, rs5988072, rs12833104, rs6318 (Cys23Ser)	No association
Kang et al., 2011 [44]	Case–control study (AP ≥ 3 months)	146 SZ	Korean	CLOZ-146, CLOZ + AP, CLOZ + NT	WC, TG, HDL, BP, GC	rs3813928 (–759C/T), rs518147 (–697G/C)	No association
Klemettilä et al., 2015 [36]	Cross-sectional (AP ≥ 1 year)	190 SZ	Europeans	CLOZ MONO-121, CLOZ + AP-69	WG, leptin, adiponectin, adipsin, IL-6, IL-1Ra, HOMA-IR, TG, HDL	rs1414334 (c.551-3008 C>G)	The GG genotype is positively associated with higher levels of TG and lower levels of HDL
Vasudev et al., 2017 [37]	Cross-sectional (AP ≥ 1 year)	60 SZ, SZA, DD	Overwhelmingly Europeans, First Nations	CLOZ-60, CLOZ MONO-18, CLOZ + AP/AD/NT	WG, WC, TG, HDL, BP, GC	rs3813929 (–759C/T), rs3813928 (–697G/A), rs1414334 (c.551-3008 C>G)	No association
Rico-Gomis et al., 2016 [45]	Cross-sectional (AP ≥ 6 months)	166 SZ, SZA, SZF, PD, BD	Europeans	CLOZ, AP	WC, TG, HDL, BP, GC	rs1414334 (c.551-3008 C>G)	No association
Puangpetch et al., 2018 [46]	Cross-sectional (AP ≥ 3 months)	113 SZ	Thais	CLOZ-47, AP, PHC	WC, TG, HDL, BP, GC	rs518147 (–697G/C), rs12836771 (A/G)—tag-SNP for rs3813929 (–759C/T)	No association
Puangpetch et al., 2019 [38]	Cross-sectional (AP long)	180 SZ	Thais	CLOZ-50, AP	GC, BMI, WC, HOMA-IR, leptin, adiponectin, prolactin, lipid profile	rs518147 (–697 G/C), rs12836771 (A/G)—tag-SNP for rs3813929 (–759C/T)	No association

Note. BD—bipolar disorder, BP—blood pressure, DD—delusional disorder, HDL—high-density lipoprotein cholesterol, NT—normothymic agent, PD—personality disorder, PHC—polypharmacy, PSD—psychotic disorder, SD—sedatives, TG—triglyceride, WC—waist circumference.

## Data Availability

All materials are presented in the article.
